# The Effect of Program Design on Engagement With an Internet-Based Smoking Intervention: Randomized Factorial Trial

**DOI:** 10.2196/jmir.2508

**Published:** 2013-03-25

**Authors:** Jennifer B McClure, Susan M Shortreed, Andy Bogart, Holly Derry, Karin Riggs, Jackie St John, Vijay Nair, Larry An

**Affiliations:** ^1^Group Health Research InstituteSeattle, WAUnited States; ^2^University of MichiganCenter for Health Communications ResearchAnn Arbor, MIUnited States; ^3^University of MichiganDepartment of StatisticsAnn Arbor, MIUnited States

**Keywords:** smoking cessation, utilization, Internet, behavioral research, electronic mail, motivation

## Abstract

**Background:**

Participant engagement influences treatment effectiveness, but it is unknown which intervention design features increase treatment engagement for online smoking cessation programs.

**Objective:**

We explored the effects of 4 design features (ie, factors) on early engagement with an Internet-based, motivational smoking cessation program.

**Methods:**

Smokers (N=1865) were recruited from a large health care organization to participate in an online intervention study, regardless of their interest in quitting smoking. The program was intended to answer smokers’ questions about quitting in an effort to motivate and support cessation. Consistent with the screening phase in the multiphase optimization strategy (MOST), we used a 2-level, full-factorial design. Each person was randomized to 1 of 2 levels of each factor, including message tone (prescriptive vs motivational), navigation autonomy (dictated vs not), proactive email reminders (yes vs no), and inclusion of personally tailored testimonials (yes vs no). The effects of each factor level on program engagement during the first 2 months of enrollment were compared, including number of visits to the website resulting in intervention content views (as opposed to supplemental content views), number of intervention content areas viewed, number of intervention content pages viewed, and duration of time spent viewing this content, as applicable to each factor.

**Results:**

Adjusting for baseline readiness to quit, persons who received content written in a prescriptive tone made the same number of visits to the website as persons receiving content in a motivational tone, but viewed 1.17 times as many content areas (95% CI 1.08-1.28; *P*<.001) and 1.15 times as many pages (95% CI 1.04-1.28; *P*=.009). Time spent viewing materials did not differ among groups (*P*=.06). Persons required to view content in a dictated order based on their initial readiness to quit made the same number of visits as people able to freely navigate the site, but viewed fewer content areas (ratio of means 0.80, 95% CI 0.74-0.87; *P*<.001), 1.17 times as many pages (95% CI 1.06-1.31; *P*=.003), and spent 1.37 times more minutes online (95% CI 1.17-1.59; *P*<.001). Persons receiving proactive email reminders made 1.20 times as many visits (95% CI 1.09-1.33; *P*<.001), viewed a similar number of content areas as persons receiving no reminders, viewed 1.58 times as many pages (95% CI 1.48-1.68; *P*<.001), and spent 1.51 times as many minutes online (95% CI 1.29-1.77; *P*<.001) as those who did not receive proactive emails. Tailored testimonials did not significantly affect engagement.

**Conclusions:**

Using a prescriptive message tone, dictating content viewing order, and sending reminder emails each resulted in greater program engagement relative to the contrasting level of each experimental factor. The results require replication, but suggest that a more directive interaction style may be preferable for online cessation programs.

**Trial Registration:**

clinicaltrials.gov NCT00992264; http://clinicaltrials.gov/ct2/show/NCT00992264 (Archived by WebCite at http://www.webcitation.org/6F7H7lr3P)

## Introduction

Smoking remains a leading cause of death and disability, accounting for approximately 1 in 5 deaths each year in the United States [1]. Effective population-based interventions are critically needed to reduce smoking prevalence and to lessen the detrimental impact of nicotine dependence. The Internet offers many advantages for this, including broad reach, low-cost treatment dissemination, and the ability to highly personalize content to be most appealing and best meet the needs of individual smokers while at the same time standardizing the content delivery across individuals (ie, to deliver personally tailored content). Based on simulation models, effective Internet-based interventions have extraordinary potential to decrease population-level smoking rates [2]. However, recent empirical reviews point out that there is only moderate evidence for the effectiveness of Internet-based cessation programs at this time [3,4]. Differences in effectiveness could be related to differences in the content or the design of existing interventions, both of which interact to dictate participants’ level of engagement with the program. Engagement has been defined as the number of site visits, number and type of pages viewed, or duration of time spent viewing the content [5-7].

Although greater program engagement does not automatically mean a program is more effective (in fact, people may not return to the program because it was effective in helping them change their behavior), some level of intervention exposure is clearly important for an intervention to have its intended effect. Research has consistently shown a dose-response effect for smoking cessation interventions, including Internet-based programs [3-5,8-12], and engagement with specific components of online programs can predict cessation [10,12,13]. But it is unclear how best to promote engagement in online nicotine dependence treatment programs in which intervention exposure is left up to the self-direction and motivation of the individual user. Evidence supports the importance of message source and the level of personal tailoring on the number of intervention pages viewed in online smoking cessation interventions [5]. Additional insight can be gleaned from studies evaluating online lifestyle modification programs. For example, supplemental email prompts can increase return website visits [14] and promote greater online self-monitoring of behavioral risk factors [15]. Others have suggested that limiting users’ control over their navigation of a website can increase time spent on the website and the number of pages visited [16]. In general, however, little is known about how to best design an Internet-based smoking cessation program to maximize participant engagement, particularly when the program is designed for use on a population level, among all smokers, regardless of their current interest in quitting. The current study addresses this issue.

Consistent with the initial screening phase of the multiphase optimization strategy (MOST) for treatment development [17,18], we implemented a 2-level full-factorial experiment to examine the effects of 4 independent design factors (message tone, navigation autonomy, proactive email outreach, and inclusion of personally tailored testimonials) on participant engagement during the first 2 months of program enrollment, with each factor explored on 2 contrasting levels. We chose to focus on the first 2 months after program enrollment because we hypothesized this to be a critical time for treatment engagement. That is, participants may be more likely to interact with the intervention shortly after joining the program, reflecting their initial motivation to participate. Future analyses will report on the long-term effects of each design factor on smoking abstinence and treatment utilization, the main outcomes for this randomized trial.

For this study, *engagement* was defined as the number of times people visited the website to view the intervention content, the number of content areas viewed, the number of content pages viewed, and the duration of time spent viewing the content. This definition is consistent with the literature [5-7] and reflects the fact that engagement is multidimensional. For instance, increased content exposure (in terms of total page views or content areas viewed) should increase one’s duration of exposure, but could also reduce the absolute number of visits if people feel they have maximized their interaction with the website. Thus, it is important to examine each of these measures separately.

Each design factor was chosen based on empirical or theoretical evidence for its effects on smoking cessation or because its treatment effects are unclear. For example, research suggests that interventions grounded in the principles of motivational interviewing can be effective across a range of health risk behaviors, including smoking abstinence [19-24]. Dictating content order based on readiness to quit may also increase treatment effectiveness by making treatment information more salient to smokers. Narrative testimonials can transport readers [25] and may result in greater behavior change [26]. In fact, personally tailored testimonials were associated with higher 6-month abstinence rates in prior research [27]. Finally, periodic email reminders may encourage greater program utilization [14,15] and, therefore, enhance treatment outcome (for further discussion of the rationale for the selection of these factors, see McClure et al [28]).

In the current study, we hypothesized that each of the experimental factor levels would also have a differential effect on our 4 measures of engagement. Participants randomized to receive online intervention content written in a prescriptive tone (as opposed to a motivational tone) would find the content less acceptable; therefore, they would view fewer content areas and Web pages, spend less time reviewing the content, and may return to the site less often. Similar hypotheses were made for people who were required to view content in a prespecified (dictated) order based on their stage of change, as opposed to being allowed to navigate the site freely, based on their interests. These assumptions are consistent with people’s desire for autonomy as described in self-determination theory [29-31]. We also believed that people who received periodic email prompts encouraging a return to the site would visit the website more often and spend more time viewing content as a result. It was unknown if they would view more treatment content areas or Web pages since exposure to the content could be maxed out during the initial visit. Finally, we explored the impact of providing smokers with personally tailored testimonials from other smokers as part of their intervention. This type of narrative is a common technique in persuasive messaging and can facilitate information processing, provide surrogate social connections, overcome resistance, and address emotional issues—all potentially important to behavior change [26]. Because the addition of the testimonials confounded our ability to examine its effects on the total number of content page views or duration of exposure (because these participants had additional content pages to view), we were only able to examine its effects on total content areas viewed and visits to the website. Findings from this study add to the nascent literature informing the optimal design of Internet-based behavior change programs to encourage program engagement.

## Methods

The study design and methods, including an extensive overview of each of the experimental factor choices, their theoretical rationale, and how each was operationalized in the Questions about Quitting (Q2) intervention is available elsewhere [28]. Details and information about the trial specific to the current hypotheses are summarized subsequently.

### Setting and Population

This study was a collaboration between Group Health Research Institute in Seattle, Washington and the University of Michigan Center for Health Communications Research in Ann Arbor, Michigan. Participants were recruited from Group Health, a large, regional nonprofit health plan in Washington State. All research materials (intervention materials, surveys, and protocols) were approved by the institutional review boards at Group Health Research Institute and the University of Michigan. The study is registered with clinicaltrials.gov (NCT00992264). Data reported in this paper were collected between May 2010 and December 2011.

### Factorial Design and Screening Experiment

Consistent with the initial phase of the MOST framework [18,32,33], we conducted a 2-level full-factorial experiment to screen for optimum intervention characteristics. Half of the participants were exposed to each contrasting level of the 4 experimental factors: message tone (prescriptive vs motivational), navigation autonomy (dictated vs not), proactive email reminders (yes vs no), and inclusion of personally tailored testimonials (yes vs no). Randomization to each factor was balanced across the trial arms to control for their effects on each factor of interest and stratified by baseline readiness to quit smoking. Interested readers are referred to McClure et al [28] for a more detailed discussion of the factorial design. Additional discussion of the MOST methodology can be found in the literature [17,18,32,33]. The long-term goal of this programmatic research will be to combine the most effective factors to create an optimized intervention and compare it to an empirically validated control in a future randomized trial.

### Recruitment, Screening, Randomization, and Enrollment

A study invitation letter was sent to adult likely smokers identified from automated health plan records. The study program was described as providing information and guidance to help people decide if quitting was right for them and how to quit if and when they decided to do so. The goal was to recruit smokers interested in quitting, as well as those with no interest in quitting.

Individuals interested in learning more about the program were provided a unique log-in access code in the invitation letter and were directed to the study website where they were screened for eligibility, provided consent, and were enrolled online. People were eligible if they were aged 18 years or older, a current member of Group Health, smoked 100 cigarettes in their lifetime, smoked even a puff in the past 7 days, smoked an average of at least 5 cigarettes per day, were not currently enrolled in a smoking cessation program or taking medication to stop smoking, had access to the Internet for personal use, were willing to check their email at least once a week, were comfortable reading and writing in English, had no visual impairments that prevented reading text on a computer screen, and were comfortable using a computer and the Internet.

After providing online consent, participants completed a baseline assessment online and then were randomized to an intervention arm using an automated algorithm. Half of all participants were randomized to each contrasting factor level and assignment to each intervention group was stratified by participants’ readiness to quit smoking at baseline (no interest in quitting in the next 6 months, interested in quitting in the next 6 months but not the next month, or interested in quitting in the next month). Following randomization, participants could immediately access their personalized intervention program following the baseline assessment and were encouraged to return to the site as often as they wanted. Because enrollment required log-in using a preassigned log-in code, it was not possible for participants to enroll in the study more than once. Participants were blinded to their group assignment.

### Program Development

The program was developed through an iterative and interactive design process. The final design and layout was informed by focus group testing with smokers. Intervention content was written by experts in behavioral science at the Group Health Research Institute and University of Michigan’s Center for Health Communications Research (CHCR). The personalized intervention content was tailored using the nonproprietary Michigan Tailoring System, developed by researchers in the CHCR. Additional detail on the program design and content are available in McClure et al [28]. There were no major changes to the intervention design or content after study launch.

### Program Design and Core Intervention Content

**Figure 1:**
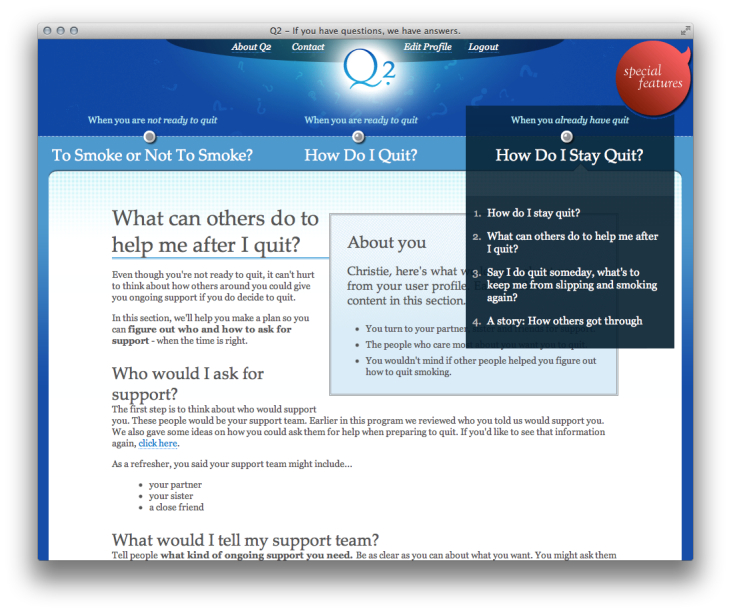
Example screenshot of Questions about Quitting (Q2) layout.

The intervention was delivered via the Internet. Participants were told they would receive an individually tailored program designed to answer their questions about quitting smoking and to help them make a decision about whether and how to stop smoking, but they were not told any specifics about the treatment arms before or after accessing the intervention. The intervention included a combination of core intervention content and additional special feature content. The core content was accessible from the main page and organized in 3 main drop-down headers or content areas, each targeting smokers at different stages of readiness to quit smoking, specifically those not ready to quit, those ready to quit, and those who already quit (see sample screenshot in Figure 1). Each of the 3 core content areas contained 3 to 5 subsections set up as individual Web pages. Section subheadings reflected questions smokers commonly have (eg, Is quitting right for me? What are my treatment options?). The special features content was also linked to the main page, but was kept distinct from the core content section. This supplemental material included topics other than smoking cessation, such as stress management, time management, and physical activity—topics thought to have a broad appeal to smokers regardless of their interest in quitting smoking and which would, therefore, encourage return visits to the website.

Participants could view the Q2 program as often as they liked and they were encouraged to return to the website in the future. Upon return, if more than 24 hours had elapsed since their last visit, participants were asked to restate their readiness to quit smoking and the content was retailored to reflect their current smoking status and interest in quitting. The basic intervention layout, number of pages, and substantive core content remained unchanged, but the text was refreshed to reflect the change in participants’ current motivation for quitting or smoking status. The intent was to ensure that the program content remained responsive to individuals’ current needs.

### Experimental Factors

#### Message Tone

Participants were randomized to receive content written in either a prescriptive or motivational tone. Prescriptive messaging was written in a didactic tone and clearly advised smokers to quit smoking and how to achieve this goal. Motivational messaging was written in a tone consistent with the key principles of motivational interviewing (express empathy, develop discrepancy, roll with resistance, support autonomy and self-efficacy) [34]. Messages written in this tone recognized smokers’ potential ambivalence about quitting and their autonomy in making decisions about if, how, and when they would quit smoking.

#### Navigation Autonomy

Half of the participants could freely view content on the website in any order they wished. The other half of the participants, in the dictated navigation arm, were required to first view content matched to their baseline readiness to quit and to view the content in a prespecified order. After this content was seen, they were then free to navigate the site.

#### Proactive Emails

Participants were also randomized to receive weekly proactive email reminders or not. Email messages were standardized across all individuals and encouraged participants to return to the Q2 website to view the optional special feature content. However, we did not track special feature page views because it was not part of the core intervention. Additionally, not all participants had access to this content at the same time. For those whose navigation of the site was dictated based on their initial readiness to quit, access to this optional content was only available after they viewed all Web pages in their initial stage-appropriate content area.

#### Testimonials

Participants were randomized to receive 3 highly tailored testimonials designed to promote their self-efficacy for quitting or to not receive these testimonials. Testimonials were tailored on each individual’s stage of change, level of nicotine dependence, prior use of pharmacotherapy for nicotine dependence, depression history, perceived risks and benefits of quitting smoking, and their self-efficacy for quitting. One testimonial was included at the end of each of the 3 core content sections. Testimonials were designed to support self-efficacy for quitting by providing personally tailored information and modeling appropriate quitting behaviors. Information was presented in an interview format with a smoker or former smoker. Because not all participants received the extra testimonial content, tracking data from these pages, including time spent viewing this content, were excluded from the analyses to normalize the engagement metrics across treatment arms.

### Assessment and Measures

Self-report data were collected at baseline using an online survey. This data included demographics; current smoking status; number of cigarettes smoked per day; stage of readiness to quit smoking; nicotine dependence assessed with the Fagerstrom Test of Nicotine Dependence [35]; motivation for quitting, self-efficacy for quitting, and perceived importance of quitting smoking (assessed on a 10-point Likert scale ranging from not at all to very); and use of alternative treatments for quitting smoking (eg, pharmacotherapy, counseling).

Automated tracking data were collected each time participants visited the website. This data included the date and time each participant visited the website and individual date/time stamps each time a content page was accessed or left.

Intervention exposure was defined as any exposure to the core intervention content. Visits to the main page and special features were excluded. Engagement was defined by: (1) the number of unique visits to the website during which the core intervention content was viewed, (2) the number of unique treatment core content sections viewed (out of a possible 3), (3) the number of times individual pages (core content subsections) were viewed, and (4) the cumulative duration of minutes spent viewing the core intervention content. Sessions automatically timed out after 30 minutes of inactivity or ended when individuals left the website (eg, logged out, closed their browser, or visited a different website).

### Data Integrity

Data were monitored over the course of the study to ensure participants were appropriately randomized, baseline data were collected, and automated user statistics on program use were being appropriately captured.

### Analyses

The analytic sample included all individuals, regardless of exposure to the core intervention content, to take advantage of the balancing effect of randomization on all covariates, measured and unmeasured. As a result, any observed differences between the randomized treatment groups are because of differences in the effect of the interventions. If we had limited the analyses to only those individuals who observed some content, this would restrict the sample based on a posttreatment outcome. That is, differences in observed program engagement levels between groups could be due to treatment effects or imbalances in covariates between the treatment groups. To further complicate matters, 1 of the factor levels (receipt of proactive emails) directly affected the probability that an individual viewed any content; limiting the analytic sample only to those who saw some content when comparing the 2 levels of this factor would bias the results.

Descriptive statistics were used to characterize the study sample based on data collected during the baseline survey. To assess engagement with the website content, we examined website-tracking data for each participant. We calculated means, standard deviations, medians, and interquartile ranges for each count-based outcome measure. We compared the number of visits to the website in which an individual viewed core intervention content between the 2 levels of each of the 4 factors using Poisson regression models that adjusted for initial readiness to quit smoking because this was a stratification variable in the randomization process. Similar Poisson models were used to estimate the effect of random factor level assignment on the number of content areas visited, ranging from zero to 3. Estimates obtained from Poisson models are generally interpretable as incidence rate ratios, but in the context of an experiment like ours in which all subjects shared a common period of exposure, estimates can equivalently be interpreted as the ratio of mean event counts among the exposed to that of the unexposed group.

The distributions of the number of individual page views and of the cumulative number of minutes spent viewing intervention content each had a larger proportion of zeros than expected from a Poisson distribution. Due to the inflated number of zeros we used zero-inflated Poisson (ZIP) models to estimate the effects of the factors on these 2 measures [36,37]. A ZIP model is made up of 2 parts: a logistic model that is used to model the excess zeros in the population and a Poisson model used to model the mean of the outcome. In this analysis, the logistic portion of each ZIP model used only an intercept to model the excess zeros for 3 of the factors. The model for the fourth factor, receiving proactive emails, included a parameter to estimate the effect of email receipt on the odds of an excess zero. The estimates reported are the effect of the factor level on the mean of the outcome (accounting for excess zeros in the corresponding Poisson distribution) in the whole population (ie, not just those who viewed the core intervention content) as described in Preisser et al [37]. No other covariate adjustments were made in the logistic portion of our ZIP models.

A total of 683 page views timed out automatically after 30 minutes of inactivity. Among page views that did not time out, most views were significantly shorter than 30 minutes, suggesting it was unlikely that all timed-out sessions truly reflected 30 minutes of time spent viewing these pages. Thus, we treated the true viewing time for these page views as missing values and imputed the viewing time for these page views using a chained equation, multiple imputation procedure [38,39]. Model predictors included baseline data (participant demographics, smoking history, beliefs about smoking, and readiness to quit), randomized level for each of the 4 factors, and the number of minutes spent on the first core content page viewed. We estimated and tested the effects of the experimental factors on the cumulative duration of intervention time by combining results from 5 imputed datasets, accounting for both within- and between-imputation variance components [40,41].

To investigate whether the effects of the random factor assignments may have differed by initial readiness to quit, we refitted each of the regression models described previously with the inclusion of interaction terms between the factors of interest and a categorical variable indicating initial readiness to quit smoking. Joint tests of the set of interaction terms within each model fit were conducted using a Wald test statistic with 2 degrees of freedom calculated to assess the significance of interactions.

Tracking data management was conducted using SAS software version 9.2 (SAS Institute, Inc, Cary, NC, USA) and all analyses, including multiple imputations, were conducted using Stata version 12 (StataCorp LP, College Station, TX, USA).

## Results

### Participants

**Figure 2:**
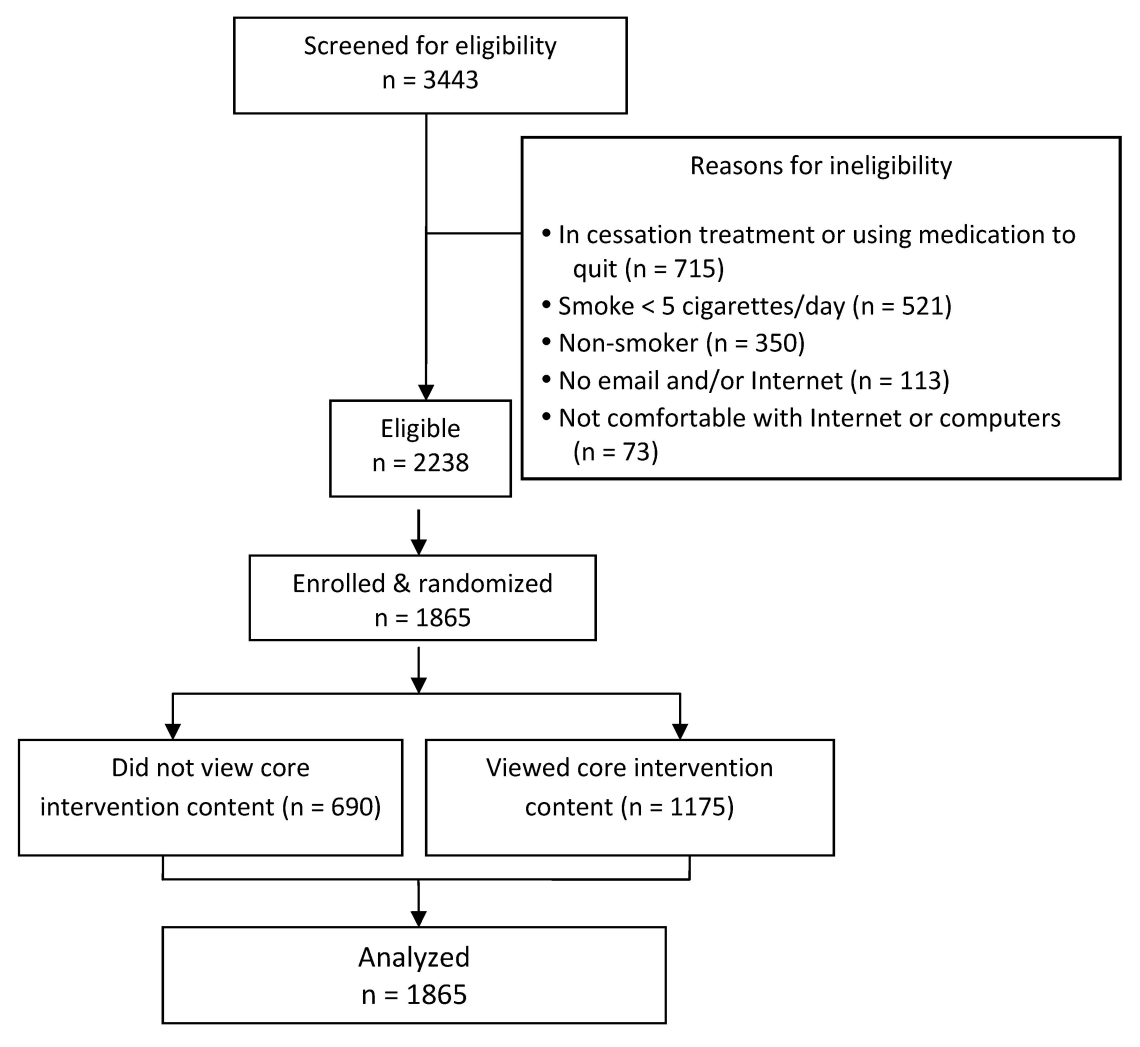
Recruitment flow and allocation of participants.

Demographic characteristics of the enrolled sample (N=1865) are presented in Table 1. The characteristics of participants within each of the 4 factors’ levels were similar to one another and to the overall distribution, so only the overall distribution is shown. Recruitment flow is presented in Figure 2. Reasons for ineligibility were not mutually exclusive. Participants could report more than 1 reason for ineligibility.

### Intervention Exposure and Engagement

#### Intervention Exposure

A total of 690 of 1865 enrolled participants (37.00%) failed to view any of the core intervention content within 2 months after joining the study, whereas 1175 participants (63.00%) viewed at least some core content during this period. Participants who failed to view any core content differed significantly with regard to their baseline readiness to quit (*P*<.001). More of these individuals had no interest in quitting smoking (15.22% vs 10.98%) or were interested in quitting in the next 6 months (46.52% vs 42.13%), but fewer were interested in quitting within the next month (38.26% vs 46.89%) indicating fewer were ready to quit smoking at baseline. Among those individuals who chose to view the core intervention, the proportion of people viewing content was similar across each factor level: message tone (64.91% prescriptive vs 61.09% motivational), navigation autonomy (64.45% dictated vs 61.55% nondictated), email reminders (63.88% yes vs 62.12% no), and testimonials (61.52% yes vs 64.48% no).

#### Program Engagement

Participants viewed the core content on a total of 1691 separate visits, resulting in 6592 unique content page views. On average, participants who accessed the core intervention made 1.4 visits (median 1, range 1-11) to view this content, viewed an average of 1.4 of the 3 core content areas (median 1, range 1-3), and viewed on average 5.6 total core content pages (median 4, range 1-53). After imputing duration of timed-out visits, the average cumulative time accrued viewing the core intervention content was 12.3 minutes (median 7.0, range 0.10-180). Of the 3 core content areas, the pages designed for people ready to quit were viewed most often. Content designed for people not yet ready to quit was viewed second most often, followed by the content designed for people who have already quit.

Engagement outcomes by factor level are presented in detail in Table 2. Effect estimates shown represent the ratio of means for each outcome measure, comparing those randomized to the stated factor level to those randomized to the contrasting factor level. For example, after adjustment for baseline readiness to quit, the average number of website visits among those who received content written in a prescriptive tone was approximately the same as the average number of visits among those whose content was written in a motivational tone, yielding a ratio of means of 1.00 (95% CI 0.90-1.10; *P*=.93). However, those viewing content in a prescriptive tone viewed an average of 1.17 times more content areas (95% CI 1.08-1.28; *P*<.001), and 1.15 times more content pages (95% CI 1.04-1.28; *P*=.009) than those whose content was written in a motivational tone. Duration of time spent viewing materials did not differ statistically between the 2 levels of the tone factor (ratio of means 0.87, 95% CI 0.75-1.01; *P*=.06). Persons receiving proactive email reminders had an average of 1.20 times as many website visits resulting in content views (95% CI 1.09-1.33; *P*<.001), but visited a similar number of content areas as persons receiving no reminders. Individuals with proactive email reminders viewed 1.58 times as many content pages (95% CI 1.48-1.68; *P*<.001), and spent 1.51 times as many minutes online (95% CI 1.29-1.77; *P*<.001). Persons required to view content in a dictated order based on their initial readiness to quit made approximately the same average number of visits as people able to freely navigate the site, but viewed fewer content areas on average (ratio of means 0.80, 95% CI 0.74-0.87; *P*<.001), viewed 1.17 times as many pages (95% CI 1.06-1.31; *P*=.003), and spent 1.37 times as many minutes online (95% CI 1.17-1.59; *P*<.001). There were no significant differences in the average number of visits to the website or content areas viewed between participants who did and did not receive the personally tailored testimonials.

**Table 1:** Baseline characteristics of enrolled participants^a^ (N=1865).

Characteristics		Participants
**Sex, n (%)**		
	Female	1178 (63.16)
**Race/ethnicity, n (%)**		
	White, non-Hispanic	1534 (82.25)
**Education level, n (%)**		
	High school or less	524 (28.10)
	Some college	944 (50.62)
	College degree or higher	396 (21.23)
**Employment status, n (%)**		
	Employed	1287 (69.00)
**Marital status, n (%)**		
	Married/partnered	1052 (56.41)
**Readiness to quit, n (%)**		
	In next 30 days	815 (43.70)
	In next 6 months, but not in next 30 days	816 (43.75)
	Not thinking of quitting	234 (12.55)
Years smoked, mean (SD)		24.9 (14.2)
Age (years), mean (SD)		44.2 (14.7)
Nicotine dependence (FTND)^b^, mean (SD)		4.2 (2.2)
**Psychosocial factors (range 1-10), mean (SD)**		
	Motivation for quitting	7.4 (2.5)
	Self-efficacy for quitting	5.4 (2.6)
	Importance of quitting	7.6 (2.6)

^a^ Complete data were available on all baseline outcomes, with 1 missing value each for race and education.

^b^ FTND: Fagerstrom Test of Nicotine Dependence.

**Table 2:** Comparison of engagement metrics by factor level.^a^

Factor level	Prescriptive message tone		Proactive emails		Dictated navigation		Tailored testimonial^b^	
	Ratio of means (95% CI)	*P*	Ratio of means (95% CI)	*P*	Ratio of means (95% CI)	*P*	Ratio of means (95% CI)	*P*
Website visits	1.00 (0.90-1.10)	.93	1.20 (1.09-1.33)	<.001	1.02 (0.92-1.13)	.70	0.92 (0.83-1.02)	.11
Content areas viewed	1.17 (1.08-1.28)	<.001	1.08 (0.99-1.17)	.10	0.80 (0.74-0.87)	<.001	0.96 (0.88-1.04)	.30
Content page views	1.15 (1.04-1.28)	.009	1.58 (1.48-1.68)	<.001	1.17 (1.06-1.31)	.003		
Cumulative duration	0.87 (0.75-1.01)	.06	1.51 (1.29-1.77)	<.001	1.37 (1.17-1.59)	<.001		

^a^ Point estimates represent ratio of means between each contrasting factor level and are adjusted for baseline stage of change. Results reflect the effect of each factor level (prescriptive tone, email reminders, dictated navigation, and testimonials) relative to those who did not receive the stated factor. Effects of randomized factors on website visits and content areas viewed were estimated with Poisson regression models, and effects on page views and duration were estimated using zero-inflated Poisson regression models.

^b^ Content page views and duration of time spent viewing content were not examined for those in the testimonial factor because these individuals had more content pages to view containing more material.

Secondary analyses investigated the interaction between baseline readiness to quit (a measure of motivation) and each of the factor levels, to determine if participants with different levels of readiness to quit at enrollment engaged differently with the core Q2 program (results not shown). Out of 17 tests for interaction, only 1 was statistically significant; the prescriptive tone resulted in significantly less cumulative viewing time (ratio of means 0.54, 95% CI 0.36-0.83) among those with no interest in quitting in the next 6 months, but had no significant effect on the viewing times of those in interested in quitting in the next month (ratio of means 0.83, 95% CI 0.66-1.04) or next 6 months (ratio of means 1.03, 95% CI 0.82-1.29). These 3 estimates, each specific to a level of readiness to quit smoking, differed significantly from one another (*P*=.02). No adjustments were made for multiple comparisons.

## Discussion

Program engagement is critical for any intervention to be effective, but promoting program engagement is a particularly important issue in Web-based interventions because treatment exposure is dependent on the motivation and self-direction of the individual user. In order to maximize the effectiveness of future Internet-based smoking treatment programs, we need a better understanding of how to engage smokers in these programs and, in particular, how to promote engagement with the most critical core program elements designed to motivate and promote behavior change. The current study provides insight into these issues by comparing the relative effects of 2 contrasting levels of each experimental design feature (factor): message tone (prescriptive vs motivational), navigation autonomy (dictated vs not), proactive email reminders (yes vs no), and inclusion of personally tailored testimonials (yes vs no). We sought to determine if one level promoted greater treatment engagement than the other within each factor.

We found that using a prescriptive message tone, dictating the order content was viewed, and sending email prompts had the greatest effects on early program engagement among a population-based sample of smokers at varying stages of readiness to quit smoking. Each of these increased the total number of core page views. Cumulative exposure to the core content was also increased by dictating navigation order and sending emails. Although the prescriptive tone was not statistically significant at the .05 level, the effect estimate was greater than 1 (*P*=.06). Using a prescriptive tone also increased the total number of core content areas viewed and, as expected, email prompts increased the number of visits to the website. No other factors increased the number of content areas viewed or number of visits made to view the core intervention content.

The inclusion of tailored testimonials did not have an effect on Web visits or the number of content areas viewed, although we did not expect it would. The primary goal of this factor was to promote smoking cessation through enhancing self-efficacy and modeling appropriate cessation-relevant behaviors, so its real impact is expected to be observed in future analyses examining long-term cessation and treatment utilization (the primary study outcomes). Also, persons whose navigation autonomy was dictated saw fewer total content areas, which was expected because they had to view all content in their stage-matched content area before gaining access to the other 2 content areas. This barrier likely deterred exposure to more content areas.

The findings suggest that using a prescriptive message tone, dictating the order in which content is viewed to match smokers’ initial level of interest in quitting, and sending weekly email prompts may increase online program engagement. This directive approach is somewhat counterintuitive for an intervention intended to motivate persons to quit smoking. Motivational interviewing suggests that people who are not ready to take action may respond better to counseling which is less directive and recognizes their ambivalence for change and autonomy to make their own decisions [34,42]. We cannot yet comment on the impact of each factor level on abstinence (the true measure of how well people respond to a cessation intervention), but in terms of program engagement, the more directive factor levels were preferable. This was unexpected for the dictated navigation, but is consistent with recently published research demonstrating that limiting user control over navigation increased time spent online and page visits within a website designed to promote hepatitis knowledge [16]. It is unclear why the prescriptive tone had a differential effect on engagement than the motivational tone—counter to what would be predicted based on self-determination theory. To gain insights into this finding we looked for differences in users’ acceptability ratings, literacy, or self-reported desire to be “told what to do” by a clinician (data not presented). These data did not reflect differences among the randomization arms that might explain our findings. The most likely explanation at this time is that smokers seeking information about whether and how to quit smoking simply prefer more directive advice. Whether this finding will generalize to other audiences or topics should be explored further.

It is noteworthy that one-third of participants failed to view any of the core intervention content during the first 2 months of the study. The reason for this is not evident, but motivation for quitting could play a role. Overall, people who failed to view core content were less likely to be ready to quit smoking in the next 30 days compared to those who viewed the content. Future planned analyses will explore how those who viewed the intervention differed from those who did not and whether these individuals failed to ever view the core intervention content or simply delayed their viewing. All participants have access to the Q2 program for a full year after enrollment.

Several caveats should be considered when interpreting these results. First, the findings might look different if we had examined tracking data for the testimonial pages and special features. However, including these would inappropriately skew the effects of the factor levels on engagement because exposure to these program elements varied by treatment arm. Also, the special feature content was not considered part of the core smoking cessation intervention. Thus, our more conservative approach is justified. The findings might also look different if engagement was observed over a longer period of time. We chose to measure engagement over the first 2 months of enrollment because this would seem to be a critical time. If one fails to engage with the program within 2 months after making an effort to enroll, it may be that they will not engage at all. We will be able to address this in future analyses when 1-year follow-up data are available. Next, the average cumulative exposure duration would be higher if we had not imputed missing values for each page view that timed out after 30 minutes of inactivity. However, we believe it preferable to treat this information as missing and use multiple imputations to accommodate this missing data in the analyses than to potentially overestimate this important outcome and artificially inflate exposure. Additionally, although it is tempting to interpret the interaction results as evidence that the prescriptive tone was less effective among people with no interest in quitting smoking, caution must be used in drawing this conclusion since this was the only significant interaction out of 17 and we did not adjust for multiple comparisons. Finally, we should caution readers not to interpret the results as an evaluation of motivational interviewing per se, which is a specific counseling technique. We can only comment on the application of several key principles of motivational interviewing when applied in a Web-based program not the full complement of motivational interviewing skills, which would be difficult to simulate outside an actual counseling session. Thus, we consider this an evaluation of a motivational message tone grounded in motivational interviewing principles.

There are limitations with this study. For one, it is not clear if the results will generalize to other Internet-based treatment programs since engagement is associated with the specific content of an intervention. But because we focused on design principles such as message tone, navigation autonomy, and use of proactive emails, it will be possible for others to apply these same strategies to future programs and test their effects. Similarly, we do not know if the results will generalize to other smokers, particularly uninsured minority males. All smokers in the current study had medical insurance (at least at the time of enrollment), most were female (63%), and most were white (82%). However, enrolling a higher proportion of female and white smokers is consistent with findings from other population-based, online cessation trials [43-45].

The study has several distinct strengths. Chief among these, the study systematically explores how the design of a public health smoking intervention influences smokers’ interaction with the program. Other strengths include the large study sample (N=1865) which included a broad spectrum of smokers with differing levels of motivation to quit, use of a rigorous study design grounded in the MOST methodological framework, use of automated tracking data to confirm individual exposure to the website at the level of each individual Web page and time spent viewing specific pages, and use of sophisticated imputation methods to account for time spent online without overinterpreting cumulative exposure time based on time-out parameters.

The results of the current study provide important insight about how to design a population-based, online smoking cessation intervention. Ultimately, it will be important to see what effect each of the experimental factors has on long-term cessation outcomes, but the current study suggests that taking a directive intervention approach, including a prescriptive message tone, dictated site navigation, and proactive email outreach may be useful for increasing program engagement particularly in population-based interventions targeting smokers with varying levels of motivation for quitting. Future research should seek to replicate these findings. Moreover, more methodologically rigorous science should seek to systematically elucidate the optimal strategies for maximizing the effectiveness of online behavioral intervention programs.

## Abbreviations

MOST: multiphase optimization strategy
Q2: Questions about Quitting (intervention)
ZIP: zero-inflated Poisson

## Multimedia Appendix 1

CONSORT-EHEALTH checklist V1.6.2 [46].

## References

[1] Centers for Disease Control and Prevention (CDC) (2002). Annual smoking-attributable mortality, years of potential life lost, and economic costs--United States, 1995-1999. MMWR Morb Mortal Wkly Rep.

[2] Levy DT, Graham AL, Mabry PL, Abrams DB, Orleans CT (2010). Modeling the impact of smoking-cessation treatment policies on quit rates. Am J Prev Med.

[3] Hutton HE, Wilson LM, Apelberg BJ, Tang EA, Odelola O, Bass EB, Chander G (2011). A systematic review of randomized controlled trials: Web-based interventions for smoking cessation among adolescents, college students, and adults. Nicotine Tob Res.

[4] Civljak M, Sheikh A, Stead LF, Car J (2010). Internet-based interventions for smoking cessation. Cochrane Database Syst Rev.

[5] Strecher VJ, McClure J, Alexander G, Chakraborty B, Nair V, Konkel J, Greene S, Couper M, Carlier C, Wiese C, Little R, Pomerleau C, Pomerleau O (2008). The role of engagement in a tailored web-based smoking cessation program: randomized controlled trial. J Med Internet Res.

[6] Danaher BG, Boles SM, Akers L, Gordon JS, Severson HH (2006). Defining participant exposure measures in Web-based health behavior change programs. J Med Internet Res.

[7] Couper MP, Alexander GL, Zhang N, Little RJ, Maddy N, Nowak MA, McClure JB, Calvi JJ, Rolnick SJ, Stopponi MA, Cole Johnson C (2010). Engagement and retention: measuring breadth and depth of participant use of an online intervention. J Med Internet Res.

[8] Fiore MC, Bailey WC, Cohen SJ, Dorfman SF, Goldstein MG, Gritz ER (2000). Clinical practice guideline: Treating tobacco use and dependence.

[9] Zbikowski SM, Jack LM, McClure JB, Deprey M, Javitz HS, McAfee TA, Catz SL, Richards J, Bush T, Swan GE (2011). Utilization of services in a randomized trial testing phone- and web-based interventions for smoking cessation. Nicotine Tob Res.

[10] An LC, Schillo BA, Saul JE, Wendling AH, Klatt CM, Berg CJ, Ahulwalia JS, Kavanaugh AM, Christenson M, Luxenberg MG (2008). Utilization of smoking cessation informational, interactive, and online community resources as predictors of abstinence: cohort study. J Med Internet Res.

[11] Elfeddali I, Bolman C, Candel MJ, Wiers RW, de Vries H (2012). Preventing smoking relapse via Web-based computer-tailored feedback: a randomized controlled trial. J Med Internet Res.

[12] Richardson A, Graham AL, Cobb N, Xiao H, Mushro A, Abrams D, Vallone D (2013). Engagement promotes abstinence in a Web-based cessation intervention: cohort study. J Med Internet Res.

[13] Schwarzer R, Satow L (2012). Online intervention engagement predicts smoking cessation. Prev Med.

[14] Schneider F, van Osch L, Schulz DN, Kremers SP, de Vries H (2012). The influence of user characteristics and a periodic email prompt on exposure to an internet-delivered computer-tailored lifestyle program. J Med Internet Res.

[15] Greaney ML, Sprunck-Harrild K, Bennett GG, Puleo E, Haines J, Viswanath KV, Emmons KM (2012). Use of email and telephone prompts to increase self-monitoring in a Web-based intervention: randomized controlled trial. J Med Internet Res.

[16] Crutzen R, Cyr D, de Vries NK (2012). The role of user control in adherence to and knowledge gained from a website: randomized comparison between a tunneled version and a freedom-of-choice version. J Med Internet Res.

[17] Collins LM, Murphy SA, Nair VN, Strecher VJ (2005). A strategy for optimizing and evaluating behavioral interventions. Ann Behav Med.

[18] Collins LM, Murphy SA, Strecher V (2007). The multiphase optimization strategy (MOST) and the sequential multiple assignment randomized trial (SMART): new methods for more potent eHealth interventions. Am J Prev Med.

[19] Burke BL, Arkowitz H, Menchola M (2003). The efficacy of motivational interviewing: a meta-analysis of controlled clinical trials. J Consult Clin Psychol.

[20] Heckman CJ, Egleston BL, Hofmann MT (2010). Efficacy of motivational interviewing for smoking cessation: a systematic review and meta-analysis. Tob Control.

[21] Hettema JE, Hendricks PS (2010). Motivational interviewing for smoking cessation: a meta-analytic review. J Consult Clin Psychol.

[22] Lundahl B, Burke BL (2009). The effectiveness and applicability of motivational interviewing: a practice-friendly review of four meta-analyses. J Clin Psychol.

[23] Vasilaki EI, Hosier SG, Cox WM (2006). The efficacy of motivational interviewing as a brief intervention for excessive drinking: a meta-analytic review. Alcohol Alcohol.

[24] Hettema J, Steele J, Miller WR (2005). Motivational interviewing. Annu Rev Clin Psychol.

[25] Green MC, Brock TC (2000). The role of transportation in the persuasiveness of public narratives. J Pers Soc Psychol.

[26] Kreuter MW, Green MC, Cappella JN, Slater MD, Wise ME, Storey D, Clark EM, O'Keefe DJ, Erwin DO, Holmes K, Hinyard LJ, Houston T, Woolley S (2007). Narrative communication in cancer prevention and control: a framework to guide research and application. Ann Behav Med.

[27] Strecher VJ, McClure JB, Alexander GL, Chakraborty B, Nair VN, Konkel JM, Greene SM, Collins LM, Carlier CC, Wiese CJ, Little RJ, Pomerleau CS, Pomerleau OF (2008). Web-based smoking-cessation programs: results of a randomized trial. Am J Prev Med.

[28] McClure JB, Derry H, Riggs KR, Westbrook EW, St John J, Shortreed SM, Bogart A, An LC (2012). Questions about quitting (Q2): design and methods of a Multiphase Optimization Strategy (MOST) randomized screening experiment for an online, motivational smoking cessation intervention. Contemp Clin Trials.

[29] Deci EL, Ryan RM (2000). The "what" and "why" of goal pursuits: human needs and the self-determination of behavior. Psychol Inquiry.

[30] Ryan RM, Deci EL (2000). Self-determination theory and the facilitation of intrinsic motivation, social development, and well-being. Am Psychol.

[31] Ryan RM, Deci EL (2006). Self-regulation and the problem of human autonomy: does psychology need choice, self-determination, and will?. J Pers.

[32] Collins LM, Baker TB, Mermelstein RJ, Piper ME, Jorenby DE, Smith SS, Christiansen BA, Schlam TR, Cook JW, Fiore MC (2011). The multiphase optimization strategy for engineering effective tobacco use interventions. Ann Behav Med.

[33] Collins LM, Dziak JJ, Li R (2009). Design of experiments with multiple independent variables: a resource management perspective on complete and reduced factorial designs. Psychol Methods.

[34] Miller WR, Rollnick S (2002). Motivational Interviewing, Second Edition: Preparing People for Change.

[35] Heatherton TF, Kozlowski LT, Frecker RC, Fagerström KO (1991). The Fagerström Test for Nicotine Dependence: a revision of the Fagerström Tolerance Questionnaire. Br J Addict.

[36] Böhning D, Dietz E, Schlattmann P, Mendonca L, Kirchner U (1999). The zero-inflated Poisson model the decayed, missing filled teeth index in dental epidemiology. J R Statist Soc A.

[37] Preisser JS, Stamm JW, Long DL, Kincade ME (2012). Review and recommendations for zero-inflated count regression modeling of dental caries indices in epidemiological studies. Caries Res.

[38] Raghunathan TE, Lepkowski JM, Hoewyk JV, Solenberger PA (2001). A multivariate technique for multiply imputing missing values using a sequence of regression models. Surv Methodol.

[39] Van Buuren S, Brand JPL, Groothuis-Oudshoorn CGM, Rubin DB (2006). Fully conditional specification in multivariate imputation. J Stat Comput Simul.

[40] Little RJA, Rubin DB (2002). Statistical Analysis with Missing Data.

[41] Raghunathan TE, Rubin DB (1991). Large-sample significance levels from multiply imputed data using moment-based statistics and an F reference distribution. J Am Stat Assoc.

[42] Rollnick S, Miller WR (2009). What is motivational interviewing?. Behav Cognit Psychother.

[43] Graham AL, Cobb NK, Papandonatos GD, Moreno JL, Kang H, Tinkelman DG, Bock BC, Niaura RS, Abrams DB (2011). A randomized trial of Internet and telephone treatment for smoking cessation. Arch Intern Med.

[44] Swan GE, McClure JB, Jack LM, Zbikowski SM, Javitz HS, Catz SL, Deprey M, Richards J, McAfee TA (2010). Behavioral counseling and varenicline treatment for smoking cessation. Am J Prev Med.

[45] Balmford J, Borland R, Li L, Ferretter I (2009). Usage of an Internet smoking cessation resource: the Australian QuitCoach. Drug Alcohol Rev.

[46] Eysenbach G, CONSORT-EHEALTH Group (2011). CONSORT-EHEALTH: improving and standardizing evaluation reports of Web-based and mobile health interventions. J Med Internet Res.

