# Supplementary material for: The Effect of Program Design on Engagement With an Internet-Based Smoking Intervention: Randomized Factorial Trial
Source: J Med Internet Res. 2013 Mar 25;15(3):e69. doi: 10.2196/jmir.2508 (PMC3636802; doi:10.2196/jmir.2508)
Supplement: Supplementary file 1 [file jmir_v15i3e69_app1.pdf]

CONSORT-EHEALTH Checklist V1.6.2 Report

(based on CONSORT-EHEALTH V1.6), available at [http://tinyurl.com/consort-ehealth-v1-6].

2508

Date completed
1/28/2013 15:59:02
by
Jennifer McClure

The effect of program design on engagement with an Internet-based smoking intervention: A randomized factorial trial

TITLE

1a-i) Identify the mode of delivery in the title
The title is, 'The effect of program design on engagement with an Internet-based smoking intervention: A randomized factorial trial.'

The term 'Internet-based' is used to reflect that the intervention included online content and email.
1a-ii) Non-web-based components or important co-interventions in title
Not applicable - the intervention was entirely Internet-based.
1a-iii) Primary condition or target group in the title
The title is, 'The effect of program design on engagement with an Internet-based smoking intervention: A randomized factorial trial.'

The title reflects that the intervention is designed for smoker and addresses smoking cessation.

ABSTRACT

1b-i) Key features/functionalities/components of the intervention and comparator in the METHODS section of the ABSTRACT
The abstract clearly identifies that we conducted a two-level, full factorial design comparing contrasting factor levels' effects on program engagement. The specific factors and their levels are also indicated. See Objective and Methods section below:

"Objectives: We explored the effects of four design features (aka, factors) on early engagement with an Internet-based, motivational smoking cessation program."

"Methods: Smokers (n = 1865) were recruited from a large healthcare organization to participate in an online intervention study, regardless of their interest in quitting smoking. The program was intended to 'answer smokers' questions about quitting', in an effort to motivate and support cessation. Consistent with the screening phase in the Multiphase Optimization Strategy (MOST), we used a two-level, full factorial design. Each person was randomized to one of two levels of each factor (message tone [prescriptive vs. motivational], navigation autonomy [dictated vs. not], proactive email reminders [yes vs. no], and inclusion of personally-tailored testimonials [yes vs. no]). The effects of each factor level on program engagement (number of visits to the website resulting in intervention content views [as opposed to supplemental content views], number of intervention content areas viewed, number of intervention content pages viewed, and duration of time spent viewing this content, as applicable to each factor) during the first two months of enrollment were compared. "
1b-ii) Level of human involvement in the METHODS section of the ABSTRACT
The abstract clearly indicates we were examining an Internet-based smoking cessation program and the intervention study was conducted online. There was no human involvement in the treatment, so none is mentioned.

"Objectives: We explored the effects of four design features (aka, factors) on early engagement with an Internet-based, motivational smoking cessation program."

"Methods: Smokers (n = 1865) were recruited from a large healthcare organization to participate in an online intervention study..."
1b-iii) Open vs. closed, web-based (self-assessment) vs. face-to-face assessments in the METHODS section of the ABSTRACT
The paper addresses each of these points. The Methods clearly state, "Adult likely smokers were identified from automated health plan records and mailed a study invitation letter... Individuals interested in learning more about the program were provided a unique log-in access code in the invitation letter and directed to the study website, where they were screened for eligibility, consented, and enrolled online." The Methods further explain the design of the intervention content and that it involved online content with proactive emails. Finally, we state that automated tracking data were used to collect engagement data and online self-report surveys were used to collect baseline data. ["Automated tracking data were collected each time participants visited the website. This data included the date and time each participant visited the website and individual date/time stamps each time a content page was accessed or left...Self-report data were collected at baseline using an online survey."]
1b-iv) RESULTS section in abstract must contain use data
Use data is provided in the abstract, as follows;
"Smokers (n = 1865) were recruited from a large healthcare organization to participate...", "Each person was randomized to one of two levels of each factor...", "Adjusting for baseline readiness to quit, persons who received content written in a prescriptive tone made the same number of visits as persons receiving content in a motivational tone, but viewed 1.17 times as many content areas (95% CI: 1.08-1.28; P <.001) and 1.15 times as many pages (95% CI: 1.04-1.28; P =.009). Time spent viewing materials did not differ between groups (P = .06). Persons required to view content in a dictated order based on their initial readiness to quit made the same number of visits as people able to freely navigate the site, but viewed fewer content areas (ratio of means = 0.80, 95% CI: 0.74-0.87; P <.001), 1.17 times as many pages (95% CI: 1.06-1.31; P =.003), and spent 1.37 times more minutes online (95% CI: 1.17-1.59;P <.001). Persons receiving proactive email reminders made 1.20 times as many visits (95% CI: 1.09-1.33; P <.001), viewed a similar number of content areas as persons receiving no reminders, viewed 1.58 times as many pages (95% CI: 1.48-1.68; P <.001), and spent 1.51 times as many minutes online (95% CI: 1.29-1.77; P < .001) as those who did not receive proactive emails. Tailored testimonials did not significantly affect engagement. "

We do not elaborate further on the use statistics in the abstract due to space constraints, but do provide additional detail in the manuscript.
1b-v) CONCLUSIONS/DISCUSSION in abstract for negative trials
This item is not relevant to the current paper. The results of this trial were not negative.

INTRODUCTION

2a-i) Problem and the type of system/solution
The Introduction outlines the problem of interest:"...little is known about how to best design an Internet-based smoking cessation program to maximize participant engagement, particularly when the program is designed for use on a population-level, among all smokers, regardless of their current interest in quitting. The current study addresses this issue. "
2a-ii) Scientific background, rationale: What is known about the (type of) system

The Introduction reviews relevant literature to online cessation programs and the effects of design features on program engagement, "Recent empirical reviews point out that there is only moderate evidence for the effectiveness of Internet-based cessation programs at this time [3, 4]. Differences in effectiveness could be related to differences in the content or the design of existing interventions, both of which interact to dictate participants' level of engagement with the program. Engagement has been defined as the number of site visits, number and type of pages viewed, or duration of time spent viewing the content [5-7]."

"While greater program engagement does not automatically mean a program is more effective (in fact, people may not return to the program because it was effective in helping them change their behavior), some level of intervention exposure is clearly important for an intervention to have its intended effect. In fact, research has consistently shown a dose-response effect for smoking cessation interventions, including Internet-based programs [3-5, 8-12] and engagement with specific components of online programs can predict cessation [10, 12, 13]. But it is unclear how best to promote engagement in online nicotine dependence treatment programs, where intervention exposure is left up to the self-direction and motivation of the individual user. Evidence supports the importance of message source and the level of personal tailoring on the number of intervention pages viewed in online smoking cessation interventions [5], and additional insight can be gleaned from studies evaluating online lifestyle modification programs. For example, supplemental email prompts can increase return website visits [14] and promote greater online self-monitoring of behavioral risk factors.[15] Others have suggested that limiting users' control over their navigation of a website can increase time spent on the website and the number of pages visited [16]."

METHODS

3a) CONSORT: Description of trial design (such as parallel, factorial) including allocation ratio

Yes, the hypotheses are clearly stated: "We hypothesized that each of the experimental factor levels would also have a differential effect on our four measures of engagement. Participants randomized to receive online intervention content written in a prescriptive tone, as opposed to a motivational tone, would find the content less acceptable and, therefore, view fewer content areas and web pages, spend less time reviewing the content, and possibly, return to the site less often. Similar hypotheses were made for people who were required to view content in a pre-specified (dictated) order based on their stage of change, as opposed to being allowed to navigate the site freely, based on their interests. These assumptions are consistent with people's desire for autonomy as described in Self-Determination Theory [28-30]. We also believed that people who received periodic email prompts encouraging a return to the site would visit the website more often and, as a result, spend more time viewing content. It was unknown if they would view more treatment content areas or web pages since exposure to the content could be maxed out during the initial visit. Finally, we explored the impact of providing smokers with personally tailored testimonials from other smokers as part of their intervention. This type of narrative is a common technique in persuasive messaging and can facilitate information processing, provide surrogate social connections, overcome resistance, and address emotional issues—all potentially important to behavior change[25]. Because the addition of the testimonials confounded our ability to examine its effects on the total number of content page views or duration of exposure (because these participants had additional content pages to view), we were only able to examine its effects on total content areas viewed and visits to the website."

3b) CONSORT: Important changes to methods after trial commencement (such as eligibility criteria), with reasons

Not applicable - we did not make any relevant changes to the study methods after enrollment began or during the period data was being collected for this study.

3b-i) Bug fixes, Downtimes, Content Changes

Not applicable - we encountered no major down times, content changes, or bug fixes after launch of this trial.

4a) CONSORT: Eligibility criteria for participants

Yes, the paper states, "People were eligible if they were 18 or older, a current member of Group Health, smoked 100 cigarettes in their lifetime, smoked even a puff in the last 7 days, averaged smoking at least 5 cigarettes per day, were not currently enrolled in a smoking cessation program or taking medication to stop smoking, had access to the Internet for personal use, were willing to check their email at least once a week, were comfortable reading and writing in English, had no visual impairments that prevented reading text on a computer screen, and were comfortable using a computer and the Internet. "

4a-i) Computer / Internet literacy

Participants were only eligible if they reported being comfortable using a computer and the Internet, as stated in the eligibility criteria above.

4a-ii) Open vs. closed, web-based vs. face-to-face assessments:

The manuscripts addresses these points, as cited in the paptper:

"Participants were recruited from Group Health, a large, regional non-profit health plan in Washington State. "

"Adult likely smokers were identified from automated health plan records and mailed a study invitation letter... Individuals interested in learning more about the program were provided a unique log-in access code in the invitation letter and directed to the study website, where they were screened for eligibility, consented, and enrolled online... Because enrollment required log-in using a pre-assigned log in code, it was not possible for participants to enroll in the study more than once... Self-report data were collected at baseline using an online survey...Automated tracking data were collected each time participants visited the website. This data included the date and time each participant visited the website and individual date/time stamps each time a content page was accessed or left."

4a-iii) Information giving during recruitment

The online consent clearly laid out the study purpose, risks, benefits, and other required consent elements. The study was presented as such, "This research study is called Questions about Quitting, or Q2. The goal of this study is to test a personalized Web-based program for people who smoke. Researchers at Group Health and the University of Michigan created this program with funds from the National Cancer Institute."

"The Q2 program provides answers to common questions about smoking and quitting smoking. It also includes helpful information about other important health issues, like managing stress. For people who are interested in quitting smoking, the program provides helpful advice on how to quit. We are testing different ways to present this content."

4b) CONSORT: Settings and locations where the data were collected

The setting and location is described as such, "This study was a collaboration between Group Health Research Institute in Seattle, Washington and the University of Michigan Center for Health Communications Research in Ann Arbor, Michigan. Participants were recruited from Group Health, a large, regional non-profit health plan in Washington State. "

4b-i) Report if outcomes were (self-)assessed through online questionnaires

Data collection for outcomes used in this study are described as such, "Self-report data were collected at baseline using an online survey...Automated tracking data were collected each time participants visited the website."

4b-ii) Report how institutional affiliations are displayed

Not applicable - the institutional affiliations were mentioned in the study invitation letter and consent, but were not prominently displayed in the online intervention.

5) CONSORT: Describe the interventions for each group with sufficient details to allow replication, including how and when they were actually administered

5-i) Mention names, credential, affiliations of the developers, sponsors, and owners

The software used in this study was developed for the study by investigators at the Group Health Research Institute and University of Michigan, as reported in the paper, " The intervention evaluated in this study was developed by researchers at the Group Health Research Institute and University of Michigan."

The software is non-proprietary and not for commercial use, so there are no conflicts of interest to report.

5-ii) Describe the history/development process

This is described in the paper as such, "The program was developed through an iterative and interactive design process. The final design and layout was informed by focus group testing with smokers. Intervention content was written by experts in behavioral science at the Group Health Research Institute and University of Michigan's Center for Health Communications Research (CHCR). The personalized intervention content was tailored using the non-proprietary Michigan Tailoring System, developed by researchers in the CHCR. Additional detail on the program design and content are available in McClure et al. [27] "

5-iii) Revisions and updating

As stated in the paper, "There were no major changes to the intervention design or content after study launch."

5-iv) Quality assurance methods

The paper states, "Data were monitored over the course of the study to ensure participants were appropriately randomized, baseline data were collected, and automated user statistics on program use were being appropriately captured."

5-v) Ensure replicability by publishing the source code, and/or providing screenshots/screen-capture video, and/or providing flowcharts of the algorithms used

We provide a detailed overview of the intervention content and design in this and our prior Methods paper (McClure et al., 2012). We also include a screenshot of the homepage which illustrates the basic layout of the intervention and sample intervention content. Additional screenshots are available, but are not published at this time since we have plans to further evaluate this program and may re-use content in future research. The source code is also preserved, but not being made public at this time.

5-vi) Digital preservation

This study is the first in a planned series of experiments which will identify important design features for online cessation programs and will culminate in the creation of an optimized intervention including the most effective design features. We have preserved the source code and detailed screenshots of the intervention, but until this research is complete, we are not making these materials publically available.

5-vii) Access

Access is described in the paper as follows, ""Participants were recruited from Group Health, a large, regional non-profit health plan in Washington State... Adult likely smokers were identified from automated health plan records and mailed a study invitation letter... Individuals interested in learning more about the program were provided a unique log-in access code in the invitation letter and directed to the study website, where they were screened for eligibility, consented, and enrolled online."

5-viii) Mode of delivery, features/functionalities/components of the intervention and comparator, and the theoretical framework

The intervention design and content was been reported on in detail in McClure et al. (2012) and is summarized in the current paper as follows:

"Consistent with the initial phase of the Multiphase Optimization Strategy (MOST) [18, 31, 32], we conducted a two-level, full factorial experiment to screen for optimum intervention characteristics. Half of the participants were exposed to each contrasting level of the four experimental factors: message tone (prescriptive vs. motivational), navigation autonomy (dictated vs. not), proactive email reminders (yes vs. no), and inclusion of personally-tailored testimonials (yes vs. no). Randomization to each factor was balanced across the trial arms to control for their effects on each factor of interest and stratified by baseline readiness to quit smoking. "

"All intervention was delivered via the internet. Participants were told they would receive an individually-tailored program designed to 'answer their questions about quitting smoking' and to help them make a decision about whether and how to stop smoking, but they were not told any specifics about the treatment arms prior to or after accessing the intervention. The intervention, called Questions about Quitting (Q2), included a combination of core intervention content and additional 'Special Feature' content. The core content was accessible from the main page and organized in three main drop-down headers or content areas, each targeting smokers at different stages of readiness to quit smoking (those not ready to quit, those ready to quit, and those already quit) (see sample screenshot in Figure 1). Each of the three core content areas contained three to five subsections, set up as individual web pages. Section sub-headings reflected questions smokers commonly have (e.g., Is quitting right for me? What are my treatment options?). The 'Special Features' content was also linked to the main page, but kept distinct from the core content section. This supplemental material included topics other than smoking cessation such as stress management, time management, and physical activity—topics thought to have a broad appeal to smokers regardless of their interest in quitting smoking and which would, therefore, encourage return visits to the website.
Participants could view the Q2 program as often as they liked and were encouraged to return to the website in the future. Upon return, if more than 24 hours had elapsed since their last visit, participants were asked to re-state their readiness to quit smoking and the content was re-tailored to reflect their current smoking status and interest in quitting. The basic intervention layout, number of pages, and substantive core content remained unchanged, but the text was refreshed to reflect the change in participants' current motivation for quitting or smoking status. The intent was to ensure that the program content remained responsive to individuals' current needs.
Experimental factors
Message tone. Participants were randomized to receive content written in either a prescriptive or motivational tone. Prescriptive messaging was written in a didactic tone and clearly advised smokers to quit smoking and how to achieve this goal. Motivational messaging was written in a tone consistent with the key principles of motivational interviewing (express empathy, develop discrepancy, roll with resistance, support autonomy and self-efficacy) [33]. Messages written in this tone recognized smokers' potential ambivalence about quitting and their autonomy in making decisions about if, how, and when they would quit smoking.
Navigation Autonomy. Half of participants could freely view content on the website in any order they wished. The other half of participants, in the dictated navigation arm, were required to first view content matched to their baseline readiness to quit and to view the content in a pre-specified order. After this content was seen, they were then free to navigate the site.
Proactive Emails. Participants were also randomized to receive weekly proactive email reminders or not. Email messages were standardized across all individuals and encouraged participants to return to the Q2 website to view the optional 'Special Feature' content. However, we did not track Special Feature page views because it was not part of the core intervention. Additionally, not all participants had access to this content at the same time. For those whose navigation of the site was dictated based on their initial readiness to quit, access to this optional content was only available after they viewed all web pages in their initial stage-appropriate content area.
Testimonials. Finally, participants were randomized to receive three highly-tailored testimonials designed to promote their self-efficacy for quitting or to not receive these testimonials. Testimonials were tailored on each individual's stage of change, level of nicotine dependence, prior use of pharmacotherapy for nicotine dependence, depression history, perceived risks and benefits of quitting smoking, and their self-efficacy for quitting. One testimonial was included at the end of each of the three core content sections. Testimonials were designed to support self-efficacy for quitting by providing personally tailored information and modeling appropriate quitting behaviors. Information was presented in an interview format with a smoker or former smoker. Because not all participants received the extra testimonial content, tracking data from these pages, including time spent viewing this content, were excluded from the analyses to normalize the engagement metrics across treatment arms. "

5-ix) Describe use parameters

Program utilization was left up to participants, althougth they were encouraged to return to the website as often as needed. The paper states, "Following randomization, participants could immediately access their personalized intervention program following the baseline assessment and were encouraged to return to the site as often as they wanted. "

5-x) Clarify the level of human involvement

As stated in the paper, "All intervention was delivered via the internet. " There was no human interaction regarding the intervention.

5-xi) Report any prompts/reminders used

Prompts are described in the study as such, "Participants were also randomized to receive weekly proactive email reminders or not. Email messages were standardized across all individuals and encouraged participants to return to the Q2 website to view the optional 'Special Feature' content."

5-xii) Describe any co-interventions (incl. training/support)

Not applicable. We did not provide any co-interventions.

6a) CONSORT: Completely defined pre-specified primary and secondary outcome measures, including how and when they were assessed

The primary outcomes in this paper were program engagement metrics. These are described in the Assessment and Measure as such, "Intervention exposure was defined as any exposure to the core intervention content. Visits to the main page and Special Features were excluded. Engagement was defined by: 1) the number of unique visits to the website during which the core intervention content was viewed, 2) the number of unique treatment core content sections viewed (out of a possible three), 3) the number of times individual pages (core content subsections) were viewed, and 4) the cumulative duration of minutes spent viewing the core intervention content. Sessions automatically timed out after 30 minutes of inactivity or ended when individuals left the website (logged out, closed their browser, or visited a different website)."

6a-i) Online questionnaires: describe if they were validated for online use and apply CHERRIES items to describe how the questionnaires were designed/deployed

No outcomes in this paper were collected using online questionnaires.

6a-ii) Describe whether and how "use" (including intensity of use/dosage) was defined/measured/monitored

As included in the paper, "Intervention exposure was defined as any exposure to the core intervention content. Visits to the main page and Special Features were excluded. Engagement was defined by: 1) the number of unique visits to the website during which the core intervention content was viewed, 2) the number of unique treatment core content sections viewed (out of a possible three), 3) the number of times individual pages (core content subsections) were viewed, and 4) the cumulative duration of minutes spent viewing the core intervention content. Sessions automatically timed out after 30 minutes of inactivity or ended when individuals left the website (logged out, closed their browser, or visited a different website). "

6a-iii) Describe whether, how, and when qualitative feedback from participants was obtained

No applicable. There is no qualitative feedback relevant to this paper.

6b) CONSORT: Any changes to trial outcomes after the trial commenced, with reasons

Not applicable - there were no changes to the trial outcomes to report.

7a) CONSORT: How sample size was determined

7a-i) Describe whether and how expected attrition was taken into account when calculating the sample size
The sample size considerations for this study have previously been described in detail in our published Methods paper (McClure et al., 2012), so they are not re-described in the current paper. Readers are referred to our prior paper for additional details on the study design and methods. In brief, the study was powered to detect a 5% difference in cessation rates at one year follow-up between people randomized to each contrasting factor level.

7b) CONSORT: When applicable, explanation of any interim analyses and stopping guidelines
We did not conduct interim analyses and there were no stopping guidelines to report.

8a) CONSORT: Method used to generate the random allocation sequence
Allocation to group assignment is described in the paper as such, "After providing online consent, participants completed a baseline assessment online and were then randomized to an intervention arm using an automated algorithm. One half of all participants were randomized to each contrasting factor level and assignment to each intervention group was stratified by participants' readiness to quit smoking at baseline (no interest in quitting in the next 6 months, interested in quitting in the next 6 months but not the next month, or interested in quitting in the next month). "

8b) CONSORT: Type of randomisation; details of any restriction (such as blocking and block size)
The randomization methods are described in the paper as such, "After providing online consent, participants completed a baseline assessment online and were then randomized to an intervention arm using an automated algorithm. One half of all participants were randomized to each contrasting factor level and assignment to each intervention group was stratified by participants' readiness to quit smoking at baseline (no interest in quitting in the next 6 months, interested in quitting in the next 6 months but not the next month, or interested in quitting in the next month). "

9) CONSORT: Mechanism used to implement the random allocation sequence (such as sequentially numbered containers), describing any steps taken to conceal the sequence until interventions were assigned
As described in the paper and noted above, we used an automated algorithm to randomize participants to groups, stratified by baseline readiness to quit smoking, to ensure balanced representation across the intervention arms. Since the randomization was automatically generated through the website, the sequence for assignment was unknown to the study team.

10) CONSORT: Who generated the random allocation sequence, who enrolled participants, and who assigned participants to interventions
As described in the paper and noted in our various responses above, the random allocation sequence was generated by an automated algorithm built into the website and which was triggered upon completion of the baseline survey. At this stage, participants were considered enrolled in the study and had access to intervention. This was a fully-automated processs.

11a) CONSORT: Blinding - If done, who was blinded after assignment to interventions (for example, participants, care providers, those assessing outcomes) and how

11a-i) Specify who was blinded, and who wasn't
Participants were blinded to group assignment. Programming staff at the Univ of Michigan and GHRI could access group assignments, but study staff at each site with no need to know this information did not have access to these files. The paper states, "Participants were blinded to their group assignment."

11a-ii) Discuss e.g., whether participants knew which intervention was the "intervention of interest" and which one was the "comparator"
This is not relevant in the current study. All participants were randomized to one of 16 different combinations of each of the 4 experimental factors of interest. Within each factor, one half of participants were randomized to each factor level, allowing each factor level to be compared to one another. This full factorial design means that no participants received a traditional "control" intervention. All received various combinations of the experimental factors of interest.

11b) CONSORT: If relevant, description of the similarity of interventions
Not relevant.

12a) CONSORT: Statistical methods used to compare groups for primary and secondary outcomes
The statistical methods are described as follows: "Descriptive statistics were used to characterize the study sample based on data collected during the baseline survey. To assess engagement with the website content, we examined website tracking data for each participant. We calculated means, standard deviations, medians, and interquartile ranges for each count-based outcome measure. We compared the number of visits to the website in which an individual viewed core intervention content between the two levels of each of the four factors using Poisson regression models which adjusted for initial readiness to quit smoking, since this was a stratification variable in the randomization process. Similar Poisson models were used to estimate the effect of random factor level assignment on the number of content areas visited, ranging from zero to three. Estimates obtained from Poisson models are generally interpretable as incidence rate ratios, but in the context of an experiment like ours, in which all subjects shared a common period of exposure, estimates can equivalently be interpreted as the ratio of mean event counts among the exposed to that of the unexposed group.
The distributions of the number of individual page views and of the cumulative number of minutes spent viewing intervention content each had a larger proportion of zeros than expected from a Poisson distribution. Due to the inflated number of zeros we fit zero-inflated Poisson (ZIP) models to estimate the effects of the factors on these two measures [35, 36]. A zero-inflated Poisson model is made up of two parts: a logistic model that is used to model the "excess" zeros in the population and a Poisson model used to model the mean of the outcome. In this analysis the logistic portion of each ZIP model used only an intercept to model the excess zeros for three of the factors. The model for the fourth factor, receiving proactive emails, included a parameter to estimate the effect of email receipt on the odds of an excess zero. The estimates reported are the effect of the factor level on the mean of the outcome (accounting for excess zeros in the corresponding Poisson distribution) in the whole population (i.e., not just those who viewed the core intervention content) as described in Preisser et al. [36]. No other covariate adjustments were made in the logistic portion of our ZIP models."

"A total of 683 page views timed out automatically after 30 minutes of inactivity. Among page views that did not time out, the majority of views were significantly shorter than 30 minutes, suggesting it was unlikely that all timed out sessions truly reflected 30 minutes of time spent viewing these pages. Thus, we treated the true viewing time for these page views as missing values and imputed the viewing time for these page views using a chained-equation, multiple imputation procedure [37, 38]. Model predictors included baseline data (participant demographics, smoking history, beliefs about smoking, and readiness to quit), randomized level for each of the four factors, and the number of minutes spent on the first core content page viewed. We estimated and tested the effects of the experimental factors on the cumulative duration of intervention time by combining results from five imputed datasets, accounting for both within- and between-imputation variance components [39, 40]."

"To investigate whether the effects of the random factor assignments may have differed by initial readiness to quit, we refit each of the regression models described above with the inclusion of interaction terms between the factors of interest and a categorical variable indicating initial readiness to quit smoking. Joint tests of the set of interaction terms within each model fit were conducted using a 2 degree-of-freedom Wald test statistic calculated to assess the significance of interactions. "

"Tracking data management was conducted using SAS software, version 9.2 [41] and all analyses, including multiple imputations, were conducted using Stata Version 12 [42]. "

12a-i) Imputation techniques to deal with attrition / missing values
Imputation techniques are described in detail in the paper, as described in the response to 12 a above.

12b) CONSORT: Methods for additional analyses, such as subgroup analyses and adjusted analyses
This is described in detail in the paper, as described in the response to 12 a above.

RESULTS

13a) CONSORT: For each group, the numbers of participants who were randomly assigned, received intended treatment, and were analysed for the primary outcome
The paper clearly outlines that half of the participants were randomized to each experimental factor level. Figure 2 depicts a CONSORT diagram, indicating the number of randomized participants, the number exposed to the intervention, and the number who were analyzed.
13b) CONSORT: For each group, losses and exclusions after randomisation, together with reasons
As depicted in the CONSORT diagram and discussed in the analyses, all randomized participants were included in the analyses.
13b-i) Attrition diagram
We do not include an attrition diagram because it is not relevant to the current analyses. We explored program engagement among all randomized participants using automated user data on website use. Participants who failed to view the website were included in the analyses, so there is no attrition in the main outcomes to be accounted for.

14a) CONSORT: Dates defining the periods of recruitment and follow-up
As reported in the paper, "Data reported in this paper were collected between May 2010 and December 2011."
14a-i) Indicate if critical "secular events" fell into the study period
There were no critical "secular events" during the study period to report.
14b) CONSORT: Why the trial ended or was stopped (early)
Not relevant - the study was not ended early.
15) CONSORT: A table showing baseline demographic and clinical characteristics for each group
This is included in Table 1.
15-i) Report demographics associated with digital divide issues
Table 1 includes gender, race, education, and employment. As outlined above, as a condition of enrollment, all participants indicated they had Internet and email access for personal use and were comfortable using the Internet.
16a) CONSORT: For each group, number of participants (denominator) included in each analysis and whether the analysis was by original assigned groups
16-i) Report multiple "denominators" and provide definitions
As reflected in Fig 2 and discussed in the analyses, all randomized participants were included in the main outcome analyses.

16-ii) Primary analysis should be intent-to-treat
As stated in the paper, "The analytic sample included all individuals, regardless of exposure to the core intervention content, to take advantage of the balancing effect of randomization on all covariates, measured and unmeasured."
17a) CONSORT: For each primary and secondary outcome, results for each group, and the estimated effect size and its precision (such as 95% confidence interval)
Effect sizes and confidence intervals are reported for each primary outcome, both in text and in Table 2.

"Engagement outcomes by factor level are presented in detail in Table 2. Effect estimates shown represent the ratio of means for each outcome measure, comparing those randomized to the stated factor level to those randomized to the contrasting factor level. For example, after adjustment for baseline readiness to quit, the average number of website visits among those who received content written in a prescriptive tone was approximately the same as the average number of visits among those whose content was written in a motivational tone, yielding a ratio of means of 1.00 (95% Confidence Interval (CI): 0.90-1.10; P=.93). However, those viewing content in a prescriptive tone viewed an average of 1.17 times more content areas (95% CI: 1.08-1.28; P <.001), and 1.15 times more content pages (95% CI: 1.04-1.28; P =.009) than those whose content was written in a motivational tone. Duration of time spent viewing materials did not differ statistically between the two levels of the tone factor (ratio of mean =0.87, 95% CI: 0.75-1.01; P = .06). Persons receiving proactive email reminders had an average of 1.20 times as many website visits resulting in content views (95% CI: 1.09-1.33; P < .001), but visited a similar number of content areas as persons receiving no reminders. Individuals with proactive email reminders viewed 1.58 times as many content pages (95% CI: 1.48-1.68; P < .001), and spent 1.51 times as many minutes online (95% CI: 1.29-1.77; P < .001). Persons required to view content in a dictated order based on their initial readiness to quit made approximately the same average number of visits as people able to freely navigate the site, but, on average, viewed fewer content areas (ratio of means = 0.80, 95% CI: 0.74-0.87; P < .001), viewed 1.17 times as many pages (95% CI: 1.06-1.31; P = .003), and spent 1.37 times as many minutes online (95% CI: 1.17-1.59; P < .001). There were no significant differences in the average number of visits to the website or content areas viewed between participants who did and did not receive the personally tailored testimonials. "
17a-i) Presentation of process outcomes such as metrics of use and intensity of use
The main outcome in this paper were the metrics of program use, as described above. Thus, we report in detail on program use for the entire sample and by randomization arm, but these are presented as main outcomes, not process measures.
17b) CONSORT: For binary outcomes, presentation of both absolute and relative effect sizes is recommended
Not applicable. We did not examine binary outcomes.
18) CONSORT: Results of any other analyses performed, including subgroup analyses and adjusted analyses, distinguishing pre-specified from exploratory
We clearly present the secondary analyses as such, "Secondary analyses investigated the interaction between baseline readiness to quit (a measure of motivation) and each of the factor levels, to determine if participants with different levels of readiness to quit at enrollment engaged differently with the core Q2 program (results not shown). "
18-i) Subgroup analysis of comparing only users
Not relevant to the current paper.
19) CONSORT: All important harms or unintended effects in each group
There were no harms or unintended adverse effects to report.
19-i) Include privacy breaches, technical problems
There were no privacy breaches or technical problems relevant to this analysis to report.
19-ii) Include qualitative feedback from participants or observations from staff/researchers
There is no qualitative feedback relevant to this paper to report.

DISCUSSION

20) CONSORT: Trial limitations, addressing sources of potential bias, imprecision, multiplicity of analyses
20-i) Typical limitations in ehealth trials

There are several caveats and trial limitations which must be considered in this paper. They are discussed in the paper as follows, "Several caveats should be considered when interpreting these results. First, the findings might look different if we had examined tracking data for the testimonial pages and Special Features. However, including these would inappropriately skew the effects of the factor levels on engagement, since exposure to these program elements varied by treatment arm. Also, the Special Feature content was not considered part of the core smoking cessation intervention. Thus, our more conservative approach is justified. The findings might also look different if engagement were observed over a longer period of time. We chose to measure engagement over the first two months of enrollment because this would seem to be a critical time. If one fails to engage with the program within two months after making an effort to enroll, it may be that they will not engage at all. We will be able to address this in future analyses when one year follow-up data are available. Next, the average cumulative exposure duration would be higher if we had not imputed missing values for each page view that timed out after 30 minutes of inactivity. However, we believe it preferable to treat this information as missing and use multiple imputation to accommodate this missing data in the analyses than to potentially over-estimate this important outcome, artificially inflating exposure. Additionally, while it is tempting to interpret the interaction results as evidence that the prescriptive tone was less effective among people with no interest in quitting smoking, caution must be used in drawing this conclusion since this was the only significant interaction out of 17 and we did not adjust for multiple comparisons. Finally, we should caution readers not to interpret the results as an evaluation of motivational interviewing per se, which is a specific counseling technique. We can only comment on the application of several key principles of motivational interviewing when applied in a web-based program, not the full complement of motivational interviewing skills, which would be difficult to simulate outside an actual counseling session. Thus, we consider this an evaluation of a motivational message tone grounded in motivational interviewing principles.
There are limitations with this study. For one, it is not clear if the results will generalize to other Internet-based treatment programs since engagement is associated with the specific content of an intervention. But because we focused on design principles such as message tone, navigation autonomy and use of proactive emails, it will be possible for others to apply these same strategies to future programs and test their effects. Similarly, we do not know if the results will generalize to other smokers, particularly uninsured, minority males. All smokers in the current study had medical insurance, at least at the time of enrollment, the majority were female (63%), and most were Caucasian (82%). However, enrolling a higher proportion of female and white smokers is consistent with findings from other population-based, online cessation trials [44-46]."

21) CONSORT: Generalisability (external validity, applicability) of the trial findings

21-i) Generalizability to other populations

The Discussion acknowledges that the findings may not generalize to other audiences or topics and that this should be explored further.

21-ii) Discuss if there were elements in the RCT that would be different in a routine application setting

Not applicable to the design or intent of the study.

22) CONSORT: Interpretation consistent with results, balancing benefits and harms, and considering other relevant evidence

22-i) Restate study questions and summarize the answers suggested by the data, starting with primary outcomes and process outcomes (use)

The overarching study question is restated in the Discussion as follows, "Program engagement is critical for any intervention to be effective, but promoting program engagement is a particularly important issue in web-based interventions because treatment exposure is dependent on the motivation and self-direction of the individual user. In order to maximize the effectiveness of future Internet-based smoking treatment programs, we need a better understanding of how to engage smokers in these programs, and in particular, how to promote engagement with the most critical core program elements designed to motivate and promote behavior change. The current study provides insight into these issues by comparing the relative effects of two contrasting levels of each experimental design feature (factor): message tone [prescriptive vs. motivational], navigation autonomy [dictated vs. not], proactive email reminders [yes vs. no], and inclusion of personally-tailored testimonials [yes vs. no]. We sought to determine whether within each factor one factor promoted greater treatment engagement or not."

22-ii) Highlight unanswered new questions, suggest future research

The results of this study suggest that using a more directive approach, including a prescriptive message tone, may increase engagement. The results for the prescriptive tone in particular are counter-intuitive based on theory. This is discussed in the paper. Additionaly, we comment that is is unknown whether "this findings will generalize to other audiences or topics", that this"should be explored," and that "future research should seek to replicate these findings."

Other information

23) CONSORT: Registration number and name of trial registry

The trial is registered, as cited in the Abstract and Methods. "The study is registered with clinicaltrials.gov (NCT00992264)."

Clinicaltrials.gov NCT00992264, http://clinicaltrials.gov/ct2/show/NCT00992264

24) CONSORT: Where the full trial protocol can be accessed, if available

Additional detail can be found in:

McClure JB, Derry H, Riggs KR, Westbrook EW, St. John J, Shortreed S, et al. Questions about quitting (Q2): Design and methods of a Multiphase Optimization Strategy (MOST) randomized screening experiment for an online, motivational smoking cessation intervention. Contemp Clin Trials. 2012; 33:1094-102.PMID:22771577.

25) CONSORT: Sources of funding and other support (such as supply of drugs), role of funders

As stated in the Acknowledgements, "This research was funded by the National Cancer Institute (R01 CA138598, J. McClure, PI)."

X26-i) Comment on ethics committee approval

The paper states, "All research materials (intervention materials, surveys, and protocols) were approved by the institutional review boards at Group Health Research Institute and the University of Michigan. "

x26-ii) Outline informed consent procedures

As stated in the paper, participants provided consent to participate online. The consent included all of the required elements including study purpose, researchers, risks, benefits, alternative intervention options, and required HIPAA language. This is not discussed in detail because it is understood that consents approved by an Institutional Review Board must include these standard required elements.

X26-iii) Safety and security procedures

Not applicable in the current study. We did not engage participants in any education or training about the safety or security of the website, although we did acknowledge the limits of online and email data privacy in the consent form.

X27-i) State the relation of the study team towards the system being evaluated

As stated in the Acknowledgements, "The intervention evaluated in this study was developed by researchers at the Group Health Research Institute and University of Michigan."
